# COVID-19 Trends and Forecast in the Eastern Mediterranean Region With a Particular Focus on Pakistan

**DOI:** 10.7759/cureus.8582

**Published:** 2020-06-12

**Authors:** Saima Dil, Nyla Dil, Zafar H Maken

**Affiliations:** 1 Animal Genomics and Biotechnology, Livestock and Dairy Development Department, Rawalpindi, PAK; 2 Medical Education: Allergy and Immunology, University of Central Florida College of Medicine, Orlando, USA; 3 Epidemiology & Public Health, Federal Medical and Dental College, Islamabad, PAK

**Keywords:** pakistan, fatality, covid-19, pandemic, coronavirus

## Abstract

First reported in China, the coronavirus responsible for coronavirus disease 2019 (COVID-19) has spread to 213 countries and territories around the world as of April 26, 2020. This study was designed to explore COVID-19 trends in the Eastern Mediterranean Region (EMR), with a particular focus on Pakistan. Daily reports and updates from the Ministry of National Health Services Regulations and Coordination COVID-19 Pakistan and the European Centre for Disease Prevention and Control were collected and study-specific data were extracted and analyzed. Our analysis revealed that, as of April 26, 2020, a total of 22 countries and territories in the EMR have reported COVID-19 cases. Iran had the highest number of cases (89,329) followed by Saudi Arabia (16,299), Pakistan (12,723), and the United Arab Emirates (9,813). Egypt (7.1%), Iran (6.3%), and Iraq (4.9%) had high case fatality rates; Lebanon (3.4%) and Pakistan (2.1%) had moderate case fatality rates; Saudi Arabia and the United Arab Emirates had low case fatality rates of 0.8% and 0.7%, respectively. Iran (76.3%) and Iraq (69.4 %) had the highest recovery rate followed by Pakistan (22.5%), the United Arab Emirates (19.2%), and Saudi Arabia (13.6%). If the current trend continues, based on the susceptible, infected, recovered (SIR) epidemiological model, we predict that EMR countries might experience a surge in the number of COVID-19 cases, resulting in as many as 2.12 million cases in Iran, 0.58 million in Saudi Arabia, and 0.51 million in Pakistan by June 20, 2020. Pakistan is the most populated country in the EMR and was the third most-affected country in terms of the number of cases with moderate case fatality and recovery rates. We predict that Pakistan’s weak healthcare system would not be able to sustain care if there is an explosive increase in the number of cases due to insufficient and inconsistent disease prevention and control policies. The best strategy for mitigating the COVID-19 pandemic is to strictly follow recommendations based on epidemiological principles.

## Introduction

Coronaviruses have previously been known for causing Severe Acute Respiratory Syndrome (SARS-CoV) in China in 2003 and Middle East Respiratory Syndrome (MERS-CoV) in Saudi Arabia in 2012. The novel virus responsible for coronavirus disease 2019 (COVID-19), termed SARS-CoV-2 (Severe Acute Respiratory Syndrome Coronavirus-2), is more contagious than SARS and MERS-CoV [1-2].

Scientific reports suggest that 81% of COVID-19 patients have a mild or asymptomatic disease, 14% have severe disease manifesting respiratory distress or pneumonia and require medical care, and 5% of hospitalized patients require transfer to intensive care units [3].

After originating from China, the novel coronavirus has spread to 213 countries and territories throughout the world as of April 26, 2020. A World Health Organization (WHO) report confirmed the first 100,000 cases in two months (December 31, 2019, to March 7, 2020), however, the number of cases increased to 200,000 in the next 12 days (March 8 to March 19, 2020) [4].

The available global data on April 26, 2020 reflects 2,804,796 COVID-19 confirmed cases with 193,710 deaths. In the Americas region, the United States of America has the highest number of confirmed cases (899,281) with 46,204 deaths, followed by Brazil with 52,995 confirmed cases and 3,670 deaths. In the Eastern Mediterranean region, Iran has reported 89,328 confirmed cases and 5,650 deaths followed by the Kingdom of Saudi Arabia with 16,299 confirmed cases and 136 deaths. Spain in the European region has reported 219,764 confirmed cases and 22,524 deaths, followed by Italy with 195,351 confirmed cases and 26,384 deaths. In the Western Pacific region, China has the highest number of confirmed cases (84,338) with 4,642 deaths, followed by Japan with 13,182 confirmed cases and 353 deaths. Reports from Southeast Asia showed 26,496 confirmed cases in India with 824 deaths, while Indonesia has reported 8,607 confirmed cases with 720 deaths. In the African region, South Africa has reported 4,361 confirmed cases with 88 deaths, and Algeria has reported 3,256 confirmed cases with 419 deaths [5]. 

The Eastern Mediterranean Region (EMR) comprises 22 countries and territories and is home to over 679 million people [6]. The region extends from Pakistan in the east to Morocco in the west, Somalia in the south, and as far north as the Islamic Republic of Iran. The region has always presented health challenges in the field of emerging zoonoses. Previous data suggest that over 60% of emerging infectious diseases in the EMR are zoonotic [7]. This region has hosted novel coronaviruses since 2012 [8].

In mid-February 2020, the first nine confirmed cases of COVID-19 were reported in the Eastern Mediterranean region. Eight cases from the United Arab Emirates tested positive between January 29 and February 9, 2020. One case from Egypt was confirmed on February 14, 2020. The Ministry of Health and Prevention of the United Arab Emirates announced that three of the reported cases recovered and were discharged from the hospital during the second week of February 2020. The confirmed case in Egypt was asymptomatic and had a history of travel to China [9]. As of April 26, 2020, the WHO has reported 160,586 confirmed COVID-19 cases with 6,887 deaths in the EMR [5].

On January 30, 2020, the WHO declared COVID-19 a Public Health Emergency of International Concern (PHEIC) [10]. Since then, countries in the Eastern Mediterranean region have been striving to enhance preparedness and taking measures to ensure early detection and rapid response [9]. The situation of COVID-19 in affected countries is changing daily. This study was designed to investigate trends of COVID-19 in the Eastern Mediterranean region with particular reference to Pakistan.

## Materials and methods

Data were collected daily from the Pakistan Ministry of National Health Services Regulations and Coordination COVID-19 dashboard and situation updates from the European Centre for Disease Prevention and Control [11-12]. The study included reports and updates from February 14 to April 26, 2020, with particular reference to Eastern Mediterranean countries. The data related to the number of cases, deaths, and recoveries were extracted from reports and recorded on spreadsheets for analysis. EMR countries with less than 90 cases on March 16, 2020 (Pakistan surpassed 100 cases on March 16, 2020) were not included in the study.

The true numbers of cases in the EMR countries were calculated based on the estimation that a substantial number of COVID-19 cases do not get reported and only 14% of the cases have been reported [13]. The number of total cases reported on April 26, 2020, was used for calculating 86% of missing true cases. The case fatality rate (CFR) and recovery rate were calculated using the following equations:

a) Case fatality rate = (total number of deaths from disease / total number of cases diagnosed with disease) x 100

b) Recovery rate = (total number of patients recovered from disease / total number of patients diagnosed with disease) x 100

The number of cases that EMR countries might expect by June 20, 2020, was estimated by using a calculator based on the susceptible, infected, recovered (SIR) model, a standard epidemiological model that is used to estimate disease dynamics [14]. These calculations were based on the following assumptions:

a) Every infected symptomatic person interacts with seven people a day

b) Every infected asymptomatic person interacts with 15 people a day 

## Results

**Figure 1:**
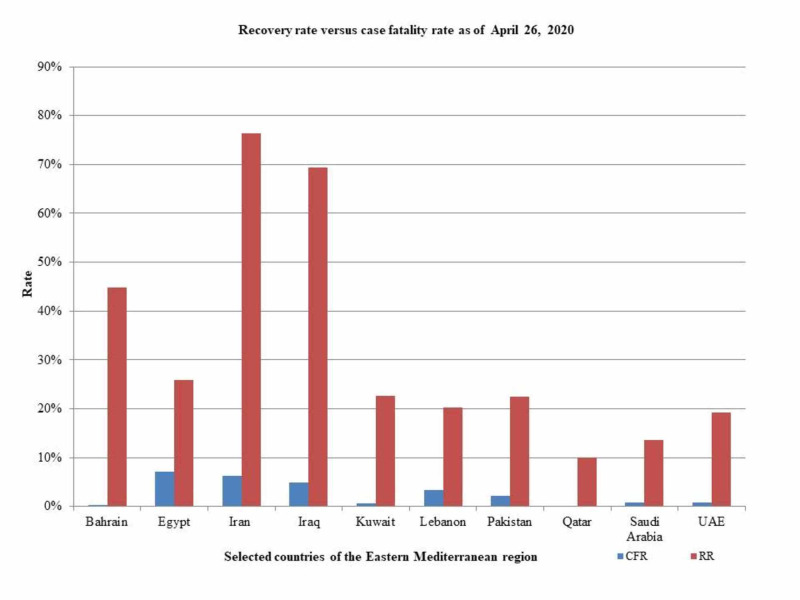
Recovery rate and case fatality rate of COVID-19 in selected countries of the Eastern Mediterranean region CFR (case fatality rate), RR (recovery rate), UAE (United Arab Emirates)

**Figure 2:**
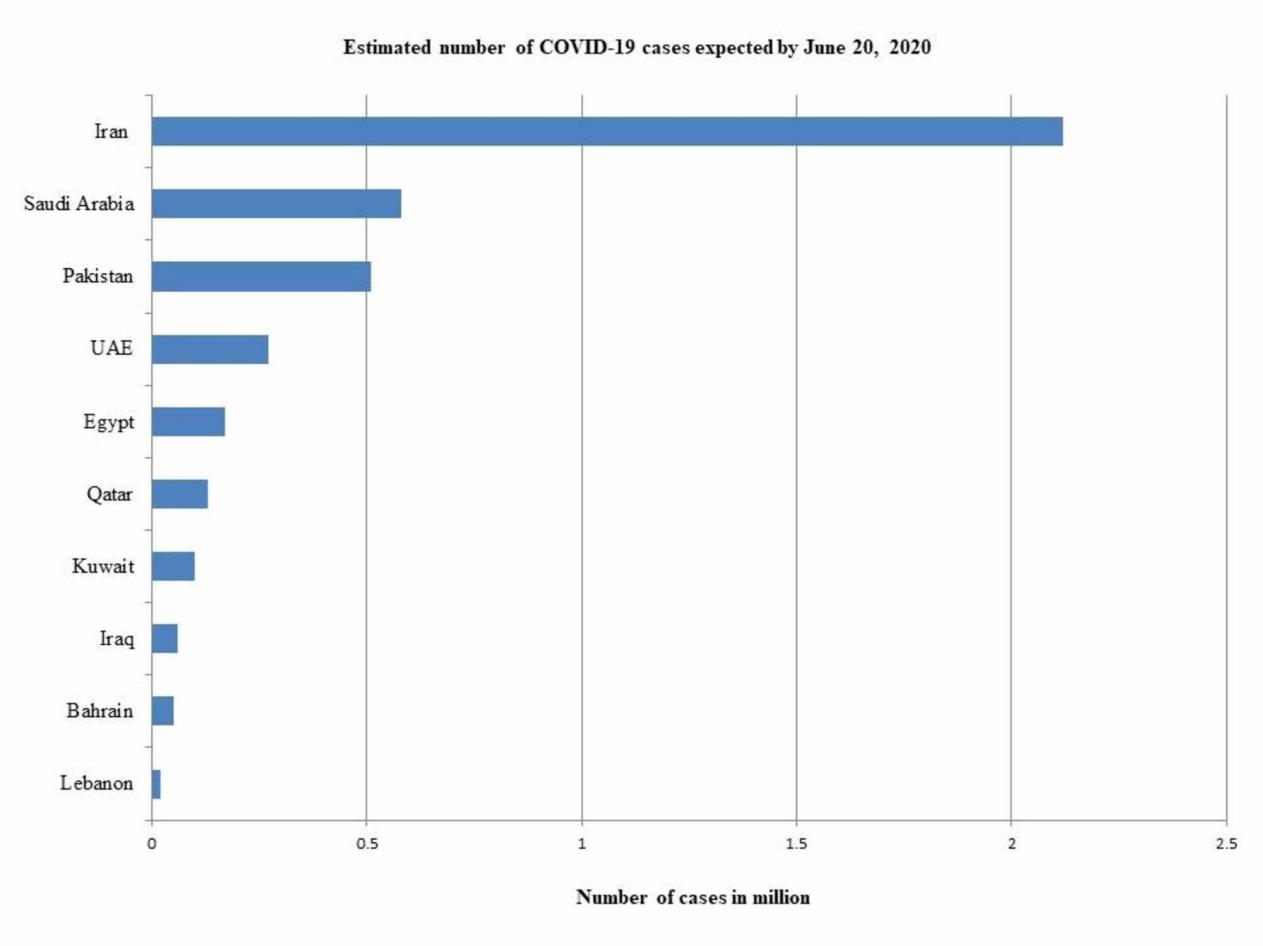
COVID-19 cases expected by June 20, 2020, in selected countries of the Eastern Mediterranean region UAE (United Arab Emirates)

As of April 26, 2020, Iran had the highest number of total of COVID-19 confirmed cases (89,329) in the EMR, with 15,485 active cases, 3,096 critical cases, 5,630 deaths, and 68,193 recoveries. The number of confirmed cases per million population was 1,064 (Table 1). The true number of cases was calculated to be 638,064 (Table 2). The doubling time for confirmed cases was determined to be 25 days. The number of deaths was doubled in 26 days. The recovery rate was calculated to be 76.3%. CFR was calculated to be 6.3% (Table 2; Figure 1). The number of cases that could be expected by June 20, 2020, was calculated to be 2.12 million (Figure 2). The number of tests per million population was 4,882 (Table 1).

**Table 1:** Confirmed case and death doubling time, recovery rate, case fatality rate, and estimated number of true cases in selected countries of the Eastern Mediterranean region as of April 26, 2020 UAE (United Arab Emirates)

Countries	Confirmed case doubling (days)	Deaths doubling time (days)	Recovery rate (%)	Case fatality rate (%)	Estimated true cases
Bahrain	10	19	44.8	0.3	18,486
Egypt	13	14	25.8	7.1	30,850
Iran	25	26	76.3	6.3	638,064
Iraq	21	27	69.4	4.9	12,593
Kuwait	10	5	22.7	0.7	20,657
Lebanon	31	24	20.3	3.4	5,029
Pakistan	11	10	22.5	2.1	90,879
Qatar	8	19	9.9	0.1	66,843
Saudi Arabia	8	12	13.6	0.8	116,421
UAE	12	11	19.2	0.7	70,093

**Table 2:** Number of COVID-19 cases, deaths, and recoveries in selected countries of the Eastern Mediterranean region as of April 26, 2020 UAE (United Arab Emirates)

Countries	Confirmed cases	Deaths	Recovered	Active cases	Critical cases	Confirmed cases/million	Test/million
Bahrain	2,588	08	1,160	1,420	02	1,521	64,869
Egypt	4,319	307	1,114	2,723	-	40	879
Iran	89,329	5,650	68,193	5650	3,096	1,064	4,882
Iraq	1,763	86	1,224	418	-	42	1,673
Kuwait	2,892	19	656	2,217	58	677	-
Lebanon	704	24	143	537	44	103	3,878
Pakistan	12,723	269	2,866	9,216	111	55	625
Qatar	9,358	10	929	8,419	72	3,248	27,665
Saudi Arabia	16,299	136	2,215	13,948	115	468	5,745
UAE	9,813	71	1,887	7,457	01	938	79,875

In Saudi Arabia, the number of confirmed COVID-19 cases was 16,299 with 13,948 active cases, 115 critical cases, 136 deaths, and 2,215 recoveries. The number of confirmed cases per million population was 468 (Table 1). The true number of cases was calculated to be 116,421 (Table 2). The number of confirmed cases was doubled in eight days. The number of deaths was doubled in 12 days. The recovery rate was calculated to be 13.6%. CFR was calculated to be 0.8% (Table 2; Figure 1). The number of cases that could be expected by June 20, 2020, was calculated to be 0.58 million (Figure 2). The number of tests per million population was 5,745 (Table 1).

In Pakistan, the number of confirmed COVID-19 cases was 12,723 with 9,216 active cases, 111 critical cases, 269 deaths, and 2,866 recoveries. The number of confirmed cases per million population was 55 (Table 1). The true number of cases was calculated to be 90,878 (Table 2). The number of confirmed cases was doubled in 11 days. The number of deaths was doubled in 10 days. The recovery rate was calculated to be 22.5%. CFR was calculated to be 2.1% (Table 2; Figure 1). The number of cases that could be expected by June 20, 2020, was calculated to be 0.51 million (Figure 2). The number of tests per million population was 625 (Table 1).

In the United Arab Emirates (UAE), the number of confirmed COVID-19 cases was 9,813, with 7,457 active cases, 71 deaths, one critical case, and 1,887 recoveries. The number of confirmed cases per million population was 938 (Table 1). The true number of cases was calculated to be 70,093 (Table 2). The number of confirmed cases was doubled in 12 days. The number of deaths was doubled in 11 days. The recovery rate was calculated to be 19.2%. CFR was calculated to be 0.7 % (Table 2; Figure 1). The number of cases that could be expected by June 20, 2020, was calculated to be 0.27 million (Figure 2). The number of tests per million population was 79,875 (Table 1).

In Qatar, the number of confirmed COVID-19 cases was 9,358, with 8,419 active cases, 72 critical cases, 10 deaths, and 929 recoveries. The number of confirmed cases per million population was 3,248 (Table 1). The true number of cases was calculated to be 66,843 (Table 2). The number of confirmed cases was doubled in eight days. The number of deaths was doubled in 10 days. The recovery rate was calculated to be 9.9%. CFR was calculated to be 0.1% (Table 2; Figure 1). The number of cases that could be expected by June 20, 2020, was calculated to be 0.13 million (Figure 2). The number of tests per million population was 27,665 (Table 1).

In Egypt, the number of confirmed COVID-19 cases was 4,319, with 2,723 active cases, 307 deaths, and 1,114 recoveries. The number of confirmed cases per million population was 40 (Table 1). The true number of cases was calculated to be 30,850 (Table 2). The number of confirmed cases was doubled in 13 days. The number of deaths was doubled in 14 days. The recovery rate was calculated to be 25.8%. CFR was calculated as 7.1 % (Table 2; Figure 1). The number of cases that could be expected by June 20, 2020, was calculated to be 0.17 million (Figure 2). The number of tests per million population was 879 (Table 1).

In Kuwait, the number of confirmed COVID-19 cases was 2,892, with 2,217 active cases, 19 deaths, 58 critical cases, and 656 recoveries. The number of confirmed cases per million population was 677 (Table 1). The true number of cases was calculated to be 20,657 (Table 2). The number of cases was doubled in 10 days. The number of deaths was doubled in five days. The recovery rate was calculated to be 22.7%. CFR was calculated to be 0.7 % (Table 2; Figure 1). The number of cases that could be expected by June 20, 2020, was calculated to be 0.1 million (Figure 2). 

In Bahrain, the number of confirmed COVID-19 was 2,588, with 1,420 active cases, eight deaths, two critical cases, and 1,160 recoveries. The number of confirmed cases per million population was 1,521 (Table 1). The true number of cases was calculated to be 30,422 (Table 2). The number of cases was doubled in 13 days. The number of deaths was doubled in 19 days. The recovery rate was calculated to be 44.8%. CFR was calculated to be 0.3 % (Table 2; Figure 1). The number of cases that could be expected by June 20, 2020, was calculated to be 0.05 million (Figure 2). The number of tests per million population was 64,869 (Table 1).

In Iraq, the number of confirmed COVID-19 was 1,763, with 418 active cases, 86 deaths, and 1,224 recoveries. The number of confirmed cases per million population was 42 (Table 1). The true number of cases was calculated to be 12,593 (Table 2). The number of cases doubled in 21 days. The number of deaths was doubled in 27 days. The recovery rate was calculated to be 69.4%. CFR was calculated to be 4.9 % (Table 2; Figure 1). The number of cases that could be expected by June 20, 2020, was calculated to be 0.06 million (Figure 2). The number of tests per million population was 1,673 (Table 1).

**Figure 3:**
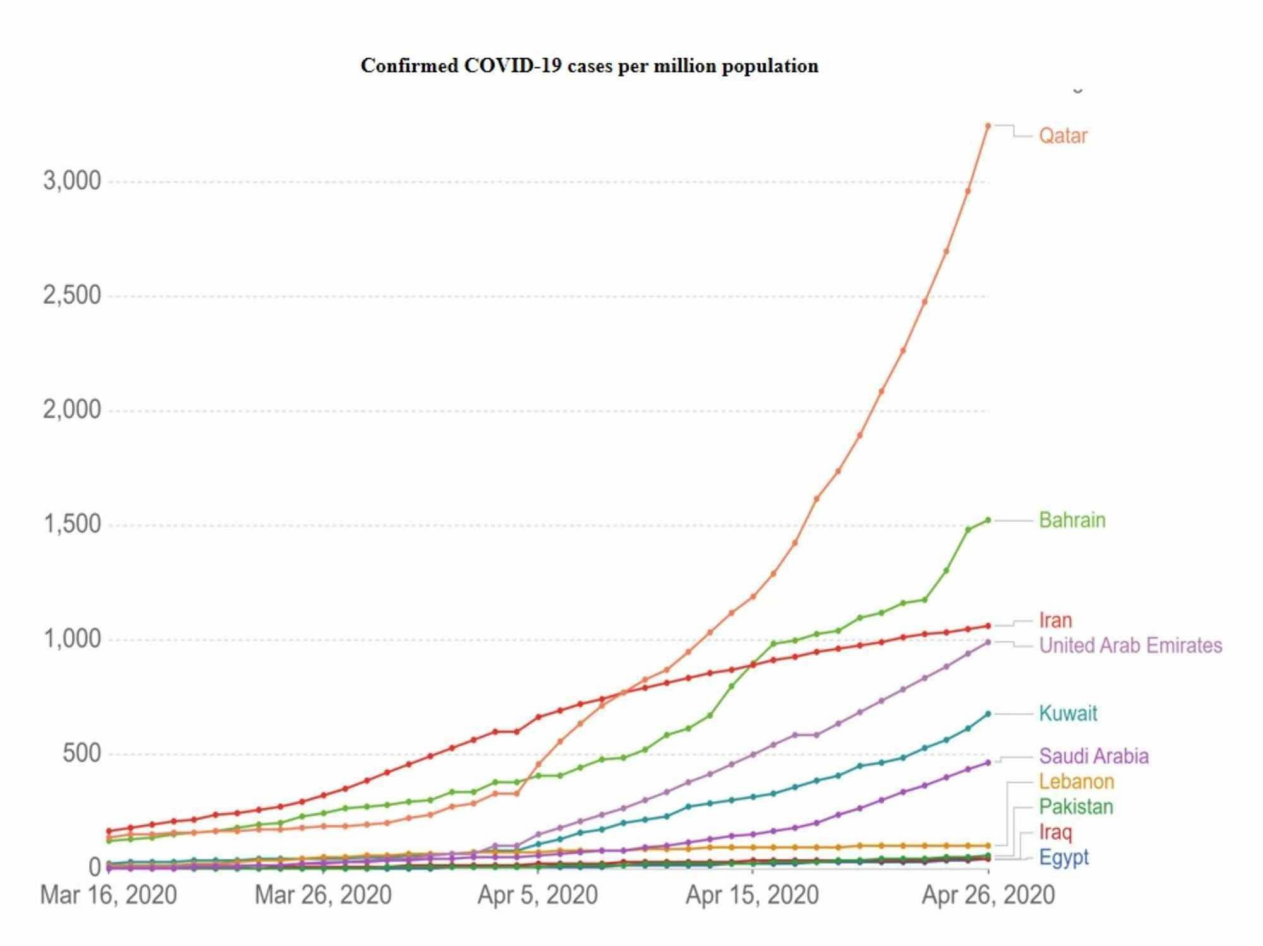
Confirmed COVID-19 cases per million population in selected countries of the Eastern Mediterranean region as of April 26, 2020 Source: European CDC - situation updated worldwide - last updated on April 26, 2020

As of April 26, 2020, Qatar had the highest number of cases per million population (3,248) followed by Bahrain (1,521), Iran (1,064), UAE (938), Kuwait (677), Saudi Arabia (468), Lebanon (103), and Pakistan (55) (Figure 3) [15].

On March 16, 2020, Pakistan surpassed 100 cases; on this date, the number of cases in Iran was 14,991, Qatar (439), Bahrain (229), Pakistan (187), Egypt (166), Saudi Arabia (133), Kuwait (130), Iraq (124), Lebanon (109), and UAE (98).

## Discussion

Pakistan, along with many countries around the world, is facing a historical public health challenge. As of April 26, 2020, Pakistan had 12,723 COVID-19 cases. In Pakistan, the COVID-19 outbreak initially presented as sporadic. Two cases were reported on February 26, 2020, and the volume of cases remained quite low (53 cases) until March 15, 2020 [16]. On March 16, 2020, the number of cases quickly rose to 187 [11]. The majority of confirmed cases presented a recent history of travel to Iran, Saudi Arabia, the United Kingdom, and Italy [17]. Since we are comparing data with other countries in the EMR, it was important to analyze the number of confirmed cases relative to the size of the respective populations (Table 1; Figure 3) [15]. The analysis showed that Pakistan had 55 cases per million population on April 26, 2020, close to Iraq (42 cases/million) and Egypt (40 cases/million) but far from Saudi Arabia (468 cases/million), the UAE (938 cases/million), and Iran (1,064 cases/million). The number of confirmed cases per million population in Pakistan was 0.03 during the first week of March 2020 and remained low (0.1) until March 14, 2020. On March 21, 2020, the number of cases/million was 2.24 and doubled (5.42) on March 28, 2020 [11]. Despite being a close neighbor to both China and Iran (two epicenters of COVID-19), Pakistan remained free of SARS-CoV-2, until mid-February 2020. This may be due to the preventive steps taken by the government of Pakistan at the beginning of the pandemic. The government of Pakistan issued a national preparedness and response plan for COVID-19 as a blueprint for pandemic preparedness for Pakistan under the Global Health Security Agenda (GHSA), after the PHEIC declaration issued by the World Health Organization [10]. This plan included instructions/standard operating protocol (SOPs) for international flight authorities, authorities at other points of entries, and health officials [18].

The number of confirmed cases was doubled in Pakistan in 11 days (Table 2), which was relatively close to that which was observed in the UAE (12 days) and Saudi Arabia (8 days), while Iran (25 days) and Iraq (21 days) had longer confirmed case doubling times. Longer confirmed case doubling times indicate a comparatively low spread of infection. Better understanding and implementation of social distancing and early detection of disease can limit the transmission of disease among the population [19]. In Pakistan, two socio-demographic factors, low literacy rate and poverty, might be big hurdles in the observance of social distancing and might have contributed to the spread of disease [20]. The literacy rate in Pakistan is 59% [21]. Most of the people do not understand the grave consequences of disease and do not follow clear instructions from the government. In Pakistan, 24.3% of the people live below the poverty line, earning their living on a day-to-day basis [22]. Therefore, a complete lockdown by the government might not be implemented. Thus, the daily interaction of symptomatic and asymptomatic COVID-19 patients might be the reason behind the rapid spread of disease.

The number of deaths doubled in Pakistan in 10 days. However, Iraq (27 days) and Iran (26 days) had a longer deaths doubling time (Table 2). In Pakistan, people may not understand the need to see a medical doctor unless they have severe symptoms. Furthermore, they rely on unqualified practitioners due to the scarcity of government-provided health services, poverty, and lack of knowledge. These factors compromise their health status and contribute to a large number of infected people seeking medical attention at a critical stage of the disease [23]. This might be the underlying reason for the shortening of the death doubling time.

The calculated case fatality rate of 2.1% for Pakistan was lower than in other countries of this region, i.e. Egypt (7.1%), Iran (6.3%), and Iraq (4.9%) (Table 2). The reason again might be that most people in Pakistan do not have access to medical facilities especially those living in remote areas and villages that might be infected but were not tested [24]. Deaths in these underprivileged areas might not be reported or documented [23]. Another possible factor is 64% of the country’s population is under 30 years of age [25]. This age group of people is very low risk for COVID-19 due to a stronger immune system that may fight pathogens more efficiently [26].

The number of true cases in Pakistan was estimated to be 90,878. The only way of knowing the exact count of confirmed COVID-19 cases depends upon the number of people tested. Pakistan, with a population of 220.9 million, is the most populated country in the EMR [27]. The number of tests performed in the country was 625/million population, which is low considering the size of the population. The number of tests/million population in the UAE (79,875) and Saudi Arabia (5,745) was significantly higher compared to Pakistan. This indicates that the actual number of COVID-19 cases might be significantly higher than the reported figures. Only 57 public laboratories in the country are conducting COVID-19 testing, and capacity is, furthermore, considerably low (30,000 tests/day), relative to the population of Pakistan [11,27]. Since only a limited number of people have access to expensive private testing facilities, a majority of people remained undiagnosed due to costs. Another reason for the low detection of COVID- 19 cases might be that 81% of patients only have mild symptoms, and patients with mild symptoms were not tested or documented [3].

An important factor linked to disease spread is the basic reproduction number (R0), which describes the average number of people who could be infected by a sick person. Currently, Pakistan has a local transmission rate of 80.6% [28]. As shown in Figure 2, the number of people who might be infected in Pakistan by June 20, 2020, could be 0.51 million. This means that Pakistan could be ranked the third highest country in the EMR after Iran (2.12 million) and Saudi Arabia (0.58 million) by June 20, 2020. A large number of COVID-19 cases will burden the threshold of medical facilities in Pakistan and might result in high mortality. According to the Economic Survey of Pakistan, there are 1,279 public sector hospitals in the country, with 220,829 registered doctors and 108,474 registered nurses. One doctor is available for 963 patients, and there is one hospital bed per 1,608 people [29]. As of now, there is no vaccine and no specific antiviral therapy for COVID-19, although several therapeutic measures are being used on an experimental basis. Supportive care is provided to relieve symptoms and maintain the function of vital organs [30]. Thus, the best strategy to fight the COVID-19 pandemic is to strictly follow recommendations based on epidemiological principles.

## Conclusions

All countries and territories in the EMR have reported cases of COVID-19, however, the number of cases and CFR varies among the EMR countries. Pakistan has the third-highest confirmed cases with moderate case fatality and recovery rates. If unswerving preventative and control measures are not adopted to prevent the widespread transmission of SARS-CoV-2, Pakistan could experience an explosive surge in the number of COVID-19 cases amounting to an estimated half a million cases by June 20, 2020, as determined by the SIR epidemiological model. This study highlights the need for strategic development and the implementation of policies and programs focused on enhanced testing, contact tracing, quarantine, and social distancing in Pakistan.
